# Ligature of the Primitive Iliac Artery

**Published:** 1842-11

**Authors:** 


*Ligature of the primitive Iliac Artery.*—The primitive iliac has been lately tied with entire success, at the Pennsylvania Hospital, by Dr. Edward Peace. We are enabled to present our readers with the following particulars of the case.

Israel Jones, a labourer, was admitted into the surgical wards of the Pennsylvania Hospital on the 22d of August, 1842, for an inguinal aneurism of five months standing. Five months previous to his entrance he strained his right groin, while lifting a heavy stone. A few days subsequently a hard tumour, about the size of a pea, made its appearance, which became as large as a walnut in the course of a month, and continued to increase until the end of the fourth month, when it attained its maximum growth. There was pulsation about the third or fourth week. About the beginning of the fourth month numbness and pain commenced in the tumour, and extended along the anterior portion of the thigh. The latter symptoms were always aggravated by exercise, and abated by rest. The man continued his daily occupations until three weeks previous to his entering the Hospital, when his sufferings became so great as to oblige him to desist. The pain at this period was so acute as to deprive him of sleep, and obliged him to maintain night and day a sitting posture, with his leg flexed on the thigh, and this on the pelvis; the whole limb resting on its exterior aspect. The tumour was large and irregular, hemispheroidal, and was, at least, two inches in height, its vertical diameter five and a half inches, and the transverse diameter about the same. It appeared to involve nearly all the right external iliac, together with some two inches of the femoral artery of the right side.

The man was an excellent subject, in the prime of life, robust, temperate, and uniformly healthy.

The operation was performed on the morning of the 29th of August, by Dr. E. Peace. assisted by his colleagues, Drs. Randolph and Norris, and Dr. J. Rhea Barton, and occupied forty-seven minutes. It was performed in the following manner:—A semi-elliptical incision was made, commencing over the anterior spinous process of the ileum nearly on a level with the umbilicus, and directed obliquely downwards to within half an inch of the external abdominal ring, and nearly parallel to Poupart's ligament. The incision was seven inches in length. The integument, the fascia of the external oblique, the external oblique, and the fascia of the internal oblique, were divided with the bistoury. The transversalis and internal oblique muscles were now exposed, and with the aponeurosis were divided on a director. The peritoneum was then separated with some difficulty, and the vessel brought into view.

The vessel was taken up about half an inch above the bifurcation, the ligature being passed around it very readily by means of Gibson's needle. Pulsation and pain ceased in the tumour the moment it was tied.

Numbness of the limb and foot, and insensibility, particularly of the toes, supervened immediately. Numbness continued to some degree, with occasional intervals, throughout the first two weeks. Sensibility gradually increased until the third day, when it was entirely restored, even in the toes. The limb below the knee became sensibly cold within an hour after the operation. It was immediately enveloped in carded wool, and recovered its natural temperature, as far down as the ankle, within the first twelve hours. At the end of twenty-four hours warmth had returned in the foot—the toes only remaining below the proper standard of heat. As the heat returned in the limb it augmented, so as to make it really warmer than the sound one, which continued to be the case during the first two weeks.

The capillary circulation in the the toes continued sluggish until the sixth day, when it appeared to be entirely restored to activity. The man experienced slight pain, with the numbness, from time to time in the affected limb, but did not suffer materially until about the middle of the second week. At this time he complained of severe pain, beginning at the toes and darting up into the tumour. This pain was relieved by the application to the tumour of lint wet with laudanum. The tumour, which had previously been rather soft, at this time became much more dense, and decidedly smaller.

No other symptom worthy of especial note presented itself, except some tumefaction of the leg, which occurred on the fifteenth day and subsided in two days.

The wound was dressed on the fourth day, and every day thereafter. The discharge was always healthy and very moderate. The man's appetite excellent, and general health improved. One-half of the wound united by the first intention; and the whole wound, except the sinus occupied by the ligature, had cicatrised within the first two weeks.

The ligature came away on September 27th, the *thirty-fifth day*. The patient is now allowed to sit up, and is doing extremely well.

The man experienced great relief from the numbness and pain, in repeated friction of the whole limb, (and especially the foot, which suffered the most in this way,) with soap liniment.

This, we believe, is the ninth time the primitive iliac has been tied. It was first tied in 1812, by Professor Gibson, of the University of Pennsylvania, for a gun-shot wound. The patient died from peritoneal inflammation on the thirteenth day. 2. By Professor Mott, in 1827. The ligature came away on the eighteenth day, and the patient recovered. 3. By Sir Phillip Crampton, in 1828. Death on the fourth day from hemorrhage. 4. By Mr. Liston, in 1829, for secondary hemorrhage after amputation. The patient, who was 8 years old, died. 5. It was tied in 1833, by Mr. Guthrie, for supposed gluteal aneurism. The operation was successful. The patient died eight months subsequently, and the disease proved to be a medullary tumour. 6. In 1837, Mr. Salomon, of St. Petersburg, tied the primitive iliac with success. 7. Mr. Syme, of Edinburgh, performed this operation in 1838. The patient died on the fourth day. 8. By M. Deguise, at the hospital of Charenton, Paris, in 1840. Successful.

Note.—Dr. Reeve, the Editor of the American Edition of Cooper's Surgical Dictionary, states that the primitive iliac was tied about six or seven years ago by Professor Bushe, in a child two months old, for congenital aneurism of one of the labia. Death occurred from abscess of the knee-joint in a few weeks.—*Philadelphia Medical Examiner.*

